# Potential Therapeutic Applications of Synthetic Conotoxin s-cal14.2b, Derived from *Californiconus californicus*, for Treating Type 2 Diabetes

**DOI:** 10.3390/biomedicines9080936

**Published:** 2021-08-01

**Authors:** Pavel H. Lugo-Fabres, Leslie M. Otero-Sastre, Johanna Bernáldez-Sarabia, Tanya A. Camacho-Villegas, Noemi Sánchez-Campos, Janeth Serrano-Bello, Luis A. Medina, Saé Muñiz-Hernández, Lizbeth de la Cruz, Isabel Arenas, Antonio Barajas-Martínez, David E. Garcia, Linda Nuñez-Garcia, Jorge González-Canudas, Alexei F. Licea-Navarro

**Affiliations:** 1 CONACYT-Unidad de Biotecnología Médica y Farmacéutica, Centro de Investigación y Asistencia en Tecnología y Diseño del Estado de Jalisco (CIATEJ) A. C., Av. Normalistas 800, Colinas de la Normal, Guadalajara 44270, Jalisco, Mexico; plugo@ciatej.mx (P.H.L.-F.); tcamacho@ciatej.mx (T.A.C.-V.); 2 Departamento de Innovación Biomédica, Centro de Investigación Científica y de Educación Superior de Ensenada (CICESE), Carretera Ensenada-Tijuana No. 3918, Zona Playitas, Ensenada 22860, Baja California, Mexico; dra.lmotero@gmail.com (L.M.O.-S.); jbernald@cicese.mx (J.B.-S.); lsanchez@cicese.edu.mx (N.S.-C.); 3 Laboratorio de Bioingeniería de Tejidos, División de Estudios de Posgrado e Investigación, Facultad de Odontología, Universidad Nacional Autónoma de México, Ciudad de México 04360, Mexico; janserbello@fo.odonto.unam.mx; 4 Laboratorio de Física Médica-Unidad de Investigación Biomédica en Cáncer-INCan, Ciudad de México 14080, Mexico; medina@fisica.unam.mx; 5 Instituto de Física, Universidad Nacional Autónoma de México (UNAM), Ciudad de México 04510, Mexico; 6 Laboratorio de Oncología Experimental, Subdirección de Investigación Básica, Instituto Nacional de Cancerología, Ciudad de México 14080, Mexico; sayide@hotmail.com; 7 Departamento de Fisiología, Facultad de Medicina, Universidad Nacional Autónoma de México, Ciudad de México 04510, Mexico; ddlc@uw.edu (L.d.l.C.); isabel.arenas@unam.mx (I.A.); antonio.barajas@c3.unam.mx (A.B.-M.); erasmo@unam.mx (D.E.G.); 8 Laboratorios Silanes S.A. de C.V., Ciudad de México 11000, Mexico; lnunez@silanes.com.mx (L.N.-G.); jogonzalez@silanes.com.mx (J.G.-C.)

**Keywords:** *Californiconus californicus*, conotoxins, s-cal14.2b, type 2 diabetes, conodrugs

## Abstract

The FDA’s approval of peptide drugs such as Ziconotide or Exendin for pain relief and diabetes treatment, respectively, enhanced the interest to explore novel conotoxins from *Conus* species venom. In general, conotoxins can be used in pathologies where voltage-gated channels, membrane receptors, or ligands alter normal physiological functions, as in metabolic diseases such as Type 2 diabetes. In this study, the synthetic cal14.2b (s-cal14.2b) from the unusual *Californiconus californicus* demonstrated bioactivity on NIT-1 insulinoma cell lines stimulating insulin secretion detecting by high performance liquid chromatography (HPLC). Accordingly, s-cal14.2b increased the Ca_V_1.2/1.3 channel-current by 35 ± 4% with a recovery τ of 10.3 ± 4 s in primary cell culture of rat pancreatic β-cells. The in vivo results indicated a similar effect of insulin secretion on mice in the glucose tolerance curve model by reducing the glucose from 500 mg/dL to 106 mg/dL in 60 min, compared to the negative control of 325 mg/dL at the same time. The PET-SCAN with radiolabeling 99mTc-s-cal14.2b demonstrated biodistribution and accumulation in rat pancreas with complete depuration in 24 h. These findings show the potential therapeutic use of s-cal14.2b in endocrinal pathologies such as early stages of Type 2 Diabetes where the pancreas’s capability to produce insulin is still effective.

## 1. Introduction

Type 2 diabetes mellitus (T2DM) is the most common endocrinological disorder worldwide. Currently, around 6.8% of the world population has the disease, increasing the number of cases in the last few decades in industrialized countries and spreading to low and medium-income countries where the microvascular complications represent an 80% mortality attributed to this disease [1,2,3]. The chronic degenerative nature of diabetes implies an economic impact associated to healthcare resources deviated to provide proper attention in the cases where complications occur such as renal diseases, amputations or blindless, and lack of productivity [2,4,5,6]. The global diabetes prevalence will rise to 578 million by 2030 and 700 million by 2045 [7,8]. The growing prevalence of childhood and adult obesity and metabolic syndrome suggests that the situation could be even worse in the next ten years [9,10]. The pathophysiology is defined as an inherited or acquired deficiency in insulin production and secretion of insulin in pancreatic β cells, or by the ineffectiveness of the insulin produced, resistance at peripheral level mainly in fat tissue, liver and muscle reflecting on high glucose blood levels damaging microvascular vessels, vision, renal function, and others [11,12,13].

Primary T2DM treatment regimens are based on sulfonylurea, biguanides, and thiazolidinediones. In the past decade, inhibitor of dipeptylpeptidase 4 (iDPP4) and sodium-glucose cotransporter-2 (SGLT-2) have been included in T2DM treatments, either monotherapy or in combination therapy, and currently represent the leading T2DM therapies [14,15,16]. However, these therapies do not prevent disease progression in most cases, and long-term administration can lead to drug tolerance and systemic toxicity [17,18,19]. The lack of T2DM treatment effectiveness can be explained by the nonspecific mechanism of action in the current therapeutics. The development of novel combinations in treatment for T2DM (for instance, metformin and incretin mimetic) focuses on the restoration of insulin sensitivity and secretion. A peptide discovered in Heloderma suspectum venom, consisting of 39 aminoacids with insulinotropic activity, called Exendin-4, is a short incretin-mimetic peptide with full agonist for the glucagon-like peptide 1 (GLP-1) receptor and produces insulinotropic effects, recently described with activity in Alzheimer disease stablish a solid precedent for T2DM therapies based on promising peptide compounds extracted from venomous animals [20,21,22,23].

In light of the worsening endocrine epidemic in developing countries (acknowledged by the American Diabetes Association (ADA) and World Health Organization (WHO), new classes of blood-glucose lowering therapeutics, such as exendin-4, are needed to enhance the effectiveness of existing treatments including lifestyle modifications, insulin, sulfonylureas, and metformin [24,25,26,27,28]. In the late 1980s, discovery of a novel analgesic in conus venom, ziconotide, began the race to discover more of these venomous compounds. Since the 1980s, the principal biomedical targets have been analgesics that act by blocking calcium or sodium channels in neurons, or by inhibiting pain impulses (i.e., prialt from Conus magus) [29,30,31,32]. In addition to ziconotide, other conotoxins are currently used as research tools in neuroscience and as potential treatments for epileptic seizures and cardiac arrest. However, limited research has focused on developing therapies from Conus toxins for treating endocrine disorders.

*Californiconus californicus* is one of the more than 800 species reported in the *Conidae* family [33]. It has its own genus (*Californiconus*), and possesses morphological characteristics associated with a comprehensive predatory behavior [34]. Geographical distribution of *C. californicus* is further north than the other Conus species. The venom from *C. californicus* is the product of at least 6 superfamily toxin genes that encode peptides composed of 8 to 50 amino acids [35,36]. Classification of Conus peptides as conotoxins depends on the abundance of cysteine residues and resulting disulfide bonds. Conotoxins are synthesized and modified by a complex enzymatic system in the venom duct where posttranslational modifications enhance the biological activities of these peptides to target membrane proteins, such as ionic channels, receptors, and other membrane proteins, and paralyze prey [35,36,37].

In the present study, for the first time, is evaluated the insulinotropic activity of synthetic conotoxin s-cal14.2b, derived from *C. californicus*, was explored in vitro using pancreatic β-cells and in vivo with animal models. Findings from this study suggest s-cal14.2b is a promising therapeutic for treating T2DM.

## 2. Materials and Methods

### 2.1. Conotoxin cal14.2b

Firstly, the native peptide of conotoxin cal14.2b was isolated from *Californiconus californicus*. It was named and reported by Biggs [33]. In this study a synthetic cal14.2b, s-cal14.2b, was from GenicBio Limited, Shanghai China. 

The native peptide of conotoxin cal14.2b was isolated from *Californiconus californicus*. Our first approach with this conotoxin was working with the fractionated venom by HPLC (data not shown). Each fraction was tested in NIT-1 insulin secretion assay and selecting the one with activity. The fraction containing the peptide of interest belongs to a family of conotoxins reported by Biggs [33]. Only cal14.2b showed in vitro potential in pancreatic β-cells. All our extended assays shown here, were achieved with the synthetic form of cal14.2b, named s-cal14.2b. The synthetic version was synthetized at GenicBio Limited, Shanghai China.

### 2.2. Isolation and Primary Culture of Metabolic Cells and NIT-1 Cell Culture

Primary cultures of metabolic cells were established from liver and pancreatic islets isolated from twelve female BALB/cAnNHsd mice (Envigo, Indianapolis, IN, USA). Mice were housed in an Optimice cage system (Animal Care Systems, Centennial, CO, USA) in a controlled environment (24 °C and 12 h light/dark cycle) and fed ad libitum with water and food (2018S Teklad Global 18% protein rodent diet, Harlan-Envigo, Indianapolis, IN, USA). Mice were acclimated for at least one week before being sacrificed according to the Guide for the Care and Use of Laboratory Animals and the Mexican Guide (NOM-062-ZOO-1999) under approval of the CICESE Bioethics Committee (CBE/PRES-O/005, 14- August 2017). The dissected liver and pancreatic tissues were treated with collagenase type I (1 mg/mL) and incubated at 37 °C for 15 min in order to detach the cells from the extracellular matrix. A 1 mL syringe plunger was gently used for mechanical disruption [38,39] Detached cells were separated and incubated in RPMI 1640 medium (Life Technologies, Grand Island, NY, USA) supplemented with 10% *v*/*v* fetal bovine serum (FBS) at 37 °C in a 5% CO_2_ atmosphere for 24 h.

The insulinoma NIT-1 cell line was from the American Type Culture Collection (CRL-2055, ATCC, Manassas, VA, USA). and grown as a model of T2DM to measure insulin secretion [40]. RPMI 1640 Cell culture media and supplements were from Sigma-Aldrich (St. Louis, MO, USA).

### 2.3. Evaluation of s-cal14.2b Cytotoxicity in NIT-1 and Primary Cell Cultures

To ensure all in vitro studies were performed with non-cytotoxic treatment concentrations of s-cal14.2b, viability of NIT-1 and primary cells was measured in response to exogenous treatment with synthetic peptide [40,41] s-cal14.2b. After three passages, 2 × 105 cells/well were grown in 96 well plates and treated with 0.1, 1, and 5 µg/mL s-cal14.2b for 24 h. Viability of NIT-1 and primary pancreatic and hepatic cells was then measured using the CellTiter 96^®^ Aqueous Cell Proliferation Assay (Promega, Madison, WI, USA). After incubating the cells with the assay reagent for 2 h at 37 °C, absorbance was measured at 490 nm using a Bio-Rad plate reader 680 Model (Bio-Rad, Hercules California, USA). The assays performed per triplicated in three independent assays.

### 2.4. NIT-1 Insulin Secretion Assay

The NIT-1 insulinoma cell line was used to measure insulin secretion in response to s-cal14.2b [42,43,44]. Cells were grown to ~80% confluence before being harvested with cold Hank’s balanced salt solution (HBSS) and supplemented with Ca^2+^ and 10% *v*/*v* FBS. The insulin stimulation assay was performed by growing 5 × 104 NIT-1 cells per well in a 96 well plate (32190102, Corning^®^) for 24 h at 37 °C in 5% CO2 before treating the cells with either 100 ng/mL s-cal14.2b, 28 mM glucose (as a positive control), or the synthetic peptide, s-cal14.1a (i.e., similar to s-cal14.2b and used as a negative control). The cells were treated for 7 min before cell media were collected for analysis. Media samples were centrifuged at 10,000× *g* with the supernatant collected and stored at −80 °C for future reverse phase high performance liquid chromatography (RP-HPLC).

The RP-HPLC analysis (Agilent 1220 Series LC System) were performed using a C18 column Varian TP54 (Palo Alto, CA, USA) with modified conditions [42]. The supernatant resulting from the insulin secretion assays were filtered in a 0.2 µm syringe filter (NACRES NB.24, Corning^®^) and charged 200 µL of medium per run. The measurement was performed at 230 nm in a linear gradient 0–35% of solvents A (0.12% (*v*/*v*) TFA and ultra-pure water) to solvent B ((*v*/*v*) of pure ACN containing 0.10% (*v*/*v*) of TFA). All solvents were obtained from Sigma-Aldrich (St. Louis, MO, USA).

### 2.5. Isolation and Culture of Rat Pancreatic β-Cells

Male Wistar rats (240–280 g) were obtained from the animal breeding facility of the School of Medicine at UNAM (Universidad Nacional Autónoma de México), and handled according to the Mexican Official Guide for Use, Care and Reproduction of Laboratory Animals (NOM-062-ZOO-1999). Experimental protocols were approved by the Institutional Ethics and Investigation Committee (identification number: 112-2013). Pancreatic β-cells were isolated from the rats [43]. Rats were anaesthetized and euthanized with an intraperitoneal sodium pentobarbital injection prior to dissection.

Pancreas insufflation employed a cold (4 °C) Hank’s Balanced Salt Solution (HBSS) with added NaHCO3 (4 mM), HEPES (15 mM), Bovine serum albumin (BSA, 1.2%) and Antibiotic-Antimycotic (100×) (1%). The pancreases were retrieved by quick dissection and placed in a tube with fresh supplemented HBSS plus Collagenase P (0.3 g/L) and heated to 37 °C in a bath for 5 min. Once enzyme digestion was finished, the pancreases were mechanically disaggregated, and the islets were handpicked under microscope. Islets clean from adjacent tissue were separated and immersed into clean supplemented HBSS. This suspension was centrifuged for 5 min at 1000× *g* rpm. Fresh supplemented HBSS was replaced and added with 1 mL of Trypsin-EDTA (2.5×) for islet dissociation in a bath at 37 °C for 3 min. The disaggregated cells were washed in RPMI medium supplemented with L-glutamine (1%), fetal bovine serum (10%), and Antibiotic-Antimycotic (100×) (1%) and decanted by centrifugation at 1000× *g* rpm for 5 min. This procedure was repeated twice. Isolated cells were incubated in a humidified atmosphere of 95% air and 5% CO_2_ at 37 °C for 16 to 24 h before patch-clamp procedures. Reagents were obtained from Sigma (St. Louis, MO, USA) unless otherwise specified. BSA was obtained from Microlab (Mexico City, Mexico), both RPMI medium and Antibiotic-Antimycotic (100×) were purchased from Life Technologies (Grand Island, NY, USA).

Pancreatic β-cells were identified by immunofluorescence. Cell cultures were fixed in 4% paraformaldehyde for 20 min, washed and then incubated for 60 min at 20 °C in PBS containing 10% bovine serum albumin and 0.3% Triton X100. Insulin goat polyclonal antibody 1:500 (Santa Cruz Biotechnology Inc., sc-7839) was incubated overnight at 4 °C. After washing out, secondary antibody AlexaFluor 546 donkey anti-goat 1:1000 (Invitrogen, A11056) was incubated at 20 °C for 2 h. FluoroShield (Abcam, ab104139) was used to mount the preparations according to the manufacturer’s instructions. As negative controls, we performed immunofluorescence using the above-mentioned protocols while omitting either the primary or secondary antibodies in parallel with the standard procedure. Cells were observed and photographed using a confocal microscope (LSM 800 Airyscan, Carl Zeiss). Zen Blue software was used to process all the images (Carl Zeiss Microscopy GmbH). Additionally, we used streptavidin–CY3 (Molecular Probes, Life Technologies, Eugene, OR, USA) to identify recording cells loaded with biotin in order to validate the capacitance range of pancreatic β-cells. In this case, β-cells were confirmed by insulin antibody (H-86)-rabbit, and anti-rabbit-FITC (Santa Cruz Biotechnology, CA, USA). Cells were observed and photographed using a confocal microscope (FV1000, Olympus, Center Valley, PA, USA).

### 2.6. Electrophysiological Analysis

Ca_V_ channel currents were recorded from rat pancreatic β-cells and by the means of the patch-clamp technique in whole-cell configuration with an EPC-9 amplifier (Patchmaster software, HEKA Electronik, Lambrecht, Germany). Recordings were taken at room temperature (22–24 °C). Currents were elicited by a voltage from −80 mV to −5 mV every 4 s. Borosilicate glass pipettes were pulled from with a patch electrode puller (Sutter Instrument, Novato, CA, USA) and filled with a solution containing 140 mM CsCl, 32 mM TEA-Cl, 10 mM HEPES, 0.1 mM BAPTA-4 Cs, 1 mM MgCl_2_, 3 mM Na_2_ATP, 3 mM Na_2_GTP, and 0.1 mM Leupeptin adjusted to pH 7.4 with CsOH. Pipette resistance was 2.5–3.5 MΩ. Cells were continuously bathed in control or test solutions with a 2 mL/min flow rate. To isolate CaV channel currents, the bath solution comprised 125 mM NaCl, 5 mM MgCl_2_, 10 mM HEPES, 10 mM l-Glucose, 10 mM BaCl_2_·2H_2_O, and 0.0001 mM TTX with the pH adjusted to 7.4 using NaOH. Series resistance was compensated to >70% and did not exceed 10 MΩ. Cell capacitance was 5–10 pF.

The s-cal14.2b (100 µg/mL) peptide was locally superfused through a large tip borosilicate pipette (3–5 μm diameter) located 20–50 μm from the cell membrane. Injection and compensation pressure were set to 250 hPa and 10 hPa respectively using an Eppendorf 5246 transjector and a 5171 micromanipulator (Eppendorf, Madison, WI, USA)

Currents were sampled at 20 kHz and filtered at 2.9 kHz. CaV channel currents were defined as the component of the current sensitive to 100 µM CdCl_2_ and using Ba^2+^ as charge carrier to enhance the Ca^2+^ channel currents trough L-type Ca^2+^ channels [44]. Steady state current amplitude was calculated as the mean value of the recorded points between 7 and 9 ms after the onset of the pulse. All values are expressed as mean ± standard error of the mean (SEM).

### 2.7. Insulinotropic Potential of s-cal14.2b In Vivo

#### 2.7.1. In Vivo Glucose Modulation by s-cal14.2b 

The effect of s-cal14.2b on in vivo glucose levels was monitored in mice with a commercial glucometer using the glucose-oxidase method. BALB/cAnNHsd mice (Har-lan-Envigo, Indianapolis, IN, USA) were housed and maintained in accordance with Bioethics Committee from CICESE (CBE/PRES-O/005). Prior to measuring glucose levels, food was removed for 12 h (with water provided ad libitum). All mice were then administered 200 µL 0.5 M glucose in PBS, by intraperitoneal injection, and divided into six experimental groups (5 mice per group (*n* = 5)) after 10 min. The positive control group received 0.1 IU recombinant insulin (Humalog^®^, Eli-Lilly Interamerica Inc. Indianapolis, IN, USA) per 20 g mouse body weight while the negative control group received sterile PBS. Four s-cal14.2b experimental groups received 65, 75, 85, or 100 µg s-cal14.2b per 20 g body weight. All mice received the different treatments by intraperitoneal injection. Blood glucose levels were measured at 0, 10, 15, 30, 60, and 120-min post-treatment. Throughout the experiment, all mice were monitored for respiratory distress and offered water ad libitum [45].

#### 2.7.2. Radiolabeling of s-cal14.2b with Technetium-99m

Technetium-99m (99mTc), a gamma-emitting radioisotope with a half-life of 6 h, was used to radiolabel s-cal14.2b in the presence of pertechnetate (TcO_4_) [46]. The labeling conjugation solution was prepared by combining 300 µg s-cal14.2b, 250 µL tartrate (2 mg/mL in acetate buffer pH5), 50 µL SnCl_2_ (2 mg/mL in 0.1 N HCL), 100 µL Gentisic acid (1 mg/mL), and 100 µL 99mTcNaTcO_4_ (3 mCi). The conjugation solution was sonicated at 80 °C for 10 min. Radiochemical purity of 99mTc-scal14.2b was evaluated by instant thin-layer chromatography on silica-impregnated glass fiber sheets (ITLC-SG) (General Electric, Santa Clara, CA, USA) using methyl ethyl ketone as the mobile phase. In vitro conjugation stability (99mTc-s-cal14.2b) was assessed 0, 1, 3, 6, and 24 h post-labeling by mixing 50 µL 99mTc-s-cal14.2b with 450 µL human serum and incubating the resulting solution at 37 °C.

#### 2.7.3. In Vivo 99mTc-s-cal14.2b Biodistribution in Normal Rats

Male Wistar rats (5 weeks old and ~250 g) were obtained from Unidad de Producción y Experimentación de Animales de Laboratorio (UPEAL-CINVESTAV-IPN, CDMX, Mexico). Animals were housed in a pathogen-free environment, maintained under controlled temperature and dark-light cycles (12 h), and fed autoclaved food and water ad libitum. The procedures for care and use of laboratory animals were approved by the institutional ethics committee (approval number: 018/052/IBI) (CI/1292/18), approved on 2018 and active at 2021. All applicable institutional and governmental regulations were in accordance with the Mexican Federal Regulations for Animal Production, Care and Experimentation (NOM-062-ZOO-1999, Ministry of Agriculture; Mexico City, Mexico). The Guide for the Care and Use of Laboratory Animals of the National Institute of Health (NIH, Bethesda, MD, USA) was also followed.

Biodistribution was measured in two experimental groups with three rats in each group (*n* = 3). The first experimental group was intravenously administered two mCi 99mTc-s-cal14.2b while the second group (control) received two mCi 99mTc. Biodistribu-tion was evaluated 1, 3, 6, 9, and 24 h after administration using an Albira image µPET/SPECT/CT system (Albira, Bruker, Spain) with next acquisition parameters CT 3D (SPECT 3D) and a field of vision encompassing the animal’s entire body (FOV). Micro-photography parameters were CT Best highly doses and high voltage (HD-HV) for all cases.

### 2.8. Statistical Analysis

All statistical analyses were performed using Prism 8.0 (GraphPad Software, Inc., San Diego, CA, USA). Data were expressed as mean ± SEM. For cell viability assays, differences between multiple groups were evaluated by one way ANOVA followed Tukey post tests. Changes in insulin secretion were analyzed by ANOVA followed by Dunnett’s post tests. Differences in glucose curve tolerance between groups were determined by *t*-test. All differences were reported as statistically significant when *p* values < 0.05.

## 3. Results

### 3.1. Cytotoxic Assesment of s-cal14.2b in NIT 1 Insulinoma and Primary Hepatic and Pancreatic Cells

**Figure 1:**
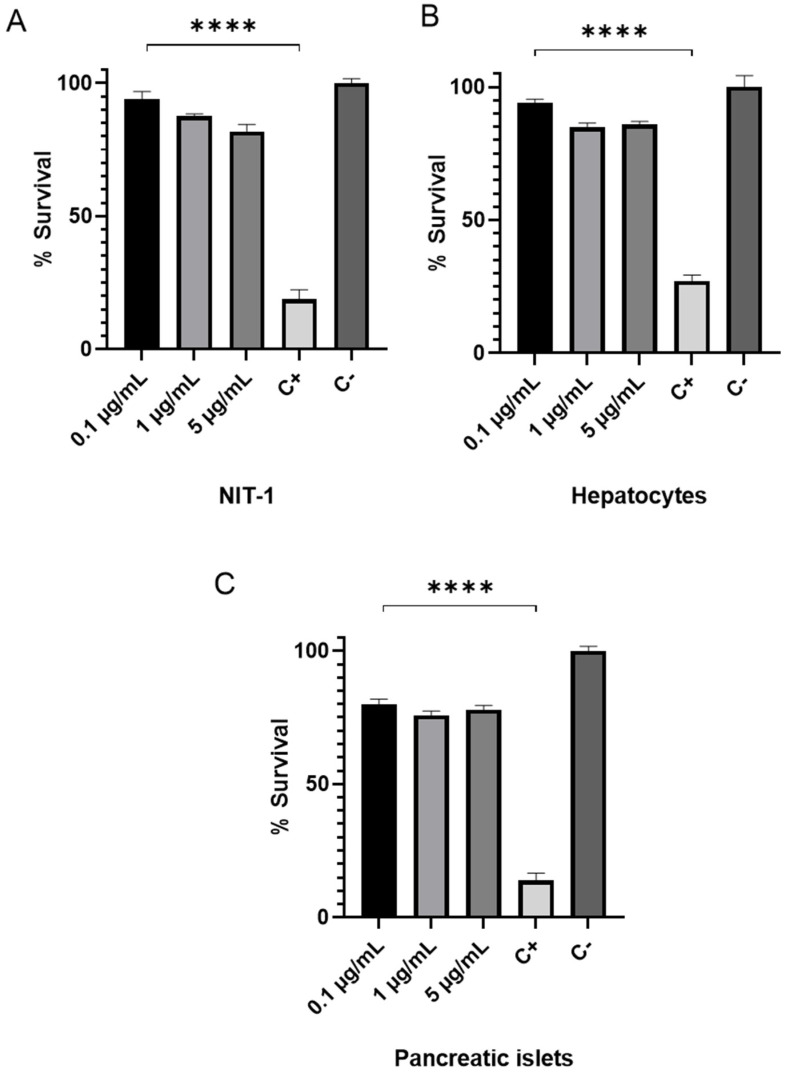
Impact of s-cal14.2b on NIT-1 and primary hepatic and pancreatic cell viability in vitro. The CellTiter 96^®^ Aqueous Cell Proliferation Assay was used to measure any changes to cell viability following treatment with 0.1, 1, and 5 µg/mL s-cal14.2b. (**A**) Cell viability of NIT-1 cells significantly decreased by 18% in response to 5 µg/mL s-cal14.2b (when compared to the negative control). (**B**) Cell viability of primary hepatocytes decreased by 15% and 14% following treatment with 1 and 5 µg/mL s-cal14.2b. (**C**) Cell viability of primary pancreatic significantly decreased by 20%, 24%, and 22% in response to 0.1, 1, and 5 µg/mL s-cal14.2b, C+ (s-cal14.1a) related peptide with cytotoxic activity, C- PBS 1x (s-cal14.2b solvent). Results are expressed mean ± SEM cell viability assays conducted in triplicate (*n* = 3). Statistical significance denoted by **** *p* < 0.0001 between control positive and s-cal14.2b concentrations.

Treatment of NIT-1 and primary hepatic and pancreatic cells with 1 and 5 µg/mL s-cal14.2b significantly decreased cell viability by ~20% compared to the negative control. However, these decreases were significantly less than the positive control that produced 70–90% decreases in cell viability. Figure 1 shows NIT-1 and primary hepatocyte cells were less affected than primary pancreatic cells probably by the heterogenic of pancreatic islets.

### 3.2. Impact of s-cal14.2b on Insulin Secretion In Vitro

**Figure 2:**
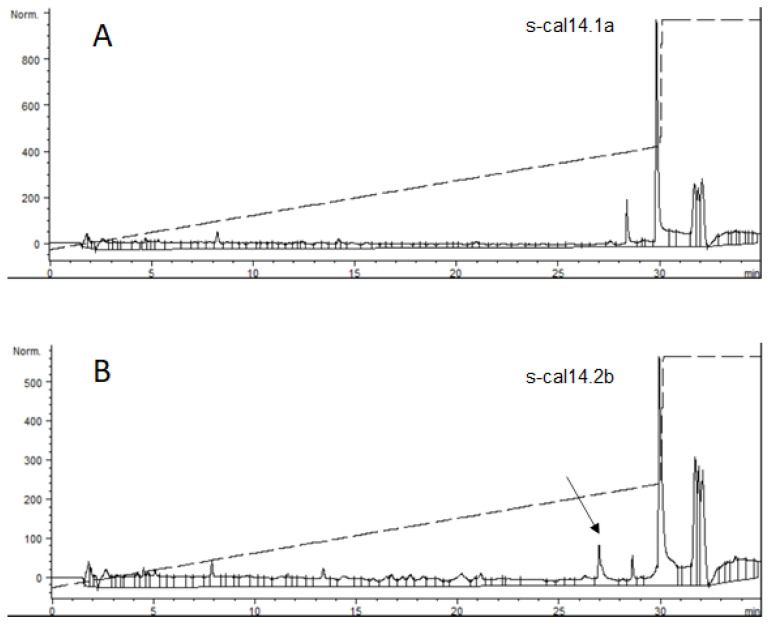
HPLC normalized chromatograms to detect insulin after (**A**) s-cal14.1a and (**B**) s-cal14.2b stimuli in NIT cells line (10 ng/mL for both peptides). The arrow indicated insulin retention time at 27 min (Rt 27), responsive to s-cal14.2b compare with the related synthetic peptide s-cal14.1a without insulin secretion effect.

Insulin secretion was measured in the media of NIT-1 cells following 24-h treatment with s-cal14.1a and s-cal14.2b. Insulin secretion was measured by RP-HPLC as shown in the chromatogram in Figure 2B. Insulin was detected with a retention time of 27 min (RT 27) in the media of NIT-1 cells treated with s-cal14.2b, but was absent in the chromatogram of media collected from NIT-1 cells treated with s-cal14.1a (Figure 2A).

### 3.3. In Vitro Immunocytochemistry Identification of Insulin in Native Rat Pancreatic β-Cells

**Figure 3:**
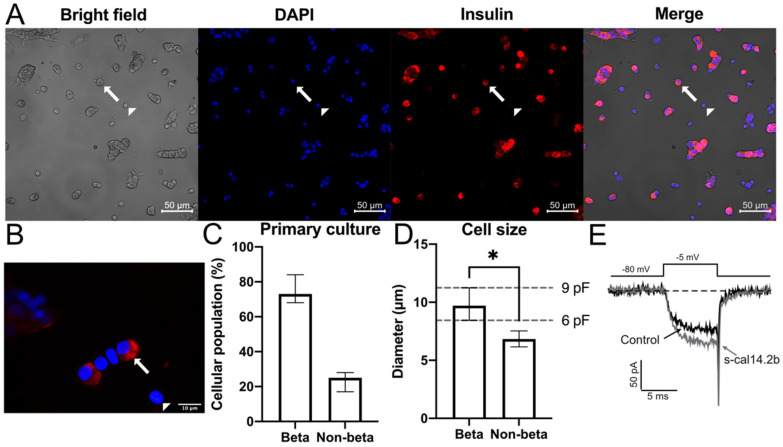
Pancreatic β-cell identification in primary cultures by immunofluorescence. (**A**) Brightfield microscopy was used to identify the cellular membrane. DAPI staining (blue) was used to identify the cellular nucleus in primary pancreatic β-cell culture at a (**A**) 40× second column and (**B**) 100× magnification. Insulin was detected (red) at (**A**) 40× third column and (**B**) 100× magnification. Cell images were merged at (**B**) 100× and (**E**) 40× magnification. The white arrow shows a typical pancreatic β-cell while the white arrowhead shows a typical non-β cell. Graphs beneath the cell image panels depict the percentage of β-cells in primary cultures (**C**) as the median ± 95% C.I. of insulin positive cells and the differences in cellular size between pancreatic β-cells and non-β cells (**D**) expressed as the median ± 95% C.I. of diameter. * indicates a difference with *p* value <0.05. The dashed lines indicate the corresponding 95% C.I. range for the recorded conductance values. For control conditions, a representative whole-cell patch-clamp calcium current in pancreatic β-cells is shown in black. The increase in calcium current induced by s-cal14.2b administration is indicated in gray.

Immunofluorescence confirmed insulin expression in primary cultures of rat pan-creatic β-cells (Figure 3A) and success of the procedure used to isolate native pancreatic β-cells [38]. Most of the cultured cells exhibited red fluorescence, making the insulin-containing region easily distinguishable from the nucleus (Figure 3B). Pancreatic β-cells also displayed significantly larger cellular diameters than non β-cells that correlated with a distinctive cellular capacitance (Figure 3D). Interestingly, these cells also exhibited a robust Ca^2+^ current, that was enhanced by s-cal14.2b (Figure 3E), and a granule-shaped appearance. Strong cell conductance was also routinely observed during calcium current measurements. Notably, Ca^2+^ current influx is a prerequisite for eliciting insulin secretion. Voltage-dependent Ca^2+^ currents are controlling β-pancreatic cells and triggering insulin secretion in response to a variety of stimuli, including elevated glucose levels.

### 3.4. Electrophysiological Assessment of Pancreatic β-Cell Ca^2+^ Currents in Response to s-cal14.2b

**Figure 4:**
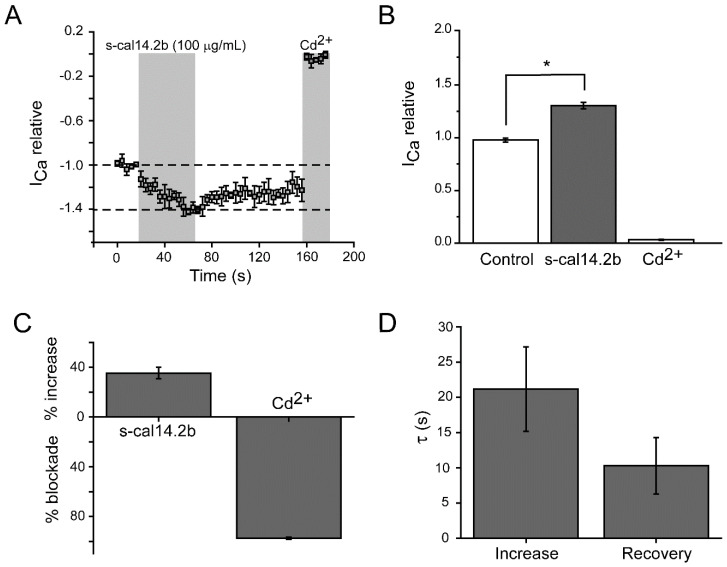
Electrophysiological assessment of pancreatic β-cell Ca2+ currents in response to s-cal14.2b. Voltage-gated calcium channel currents (Cav1.2/1.3) were recorded in response to s-cal14.2b using the patch-clamp technique in whole-cell configuration. (**A**) Time-course of Ca^2+^ current in β-cells following 100 µg/mL s-cal14.2b microperfusion. The gray area indicates application time of s-cal14.2b or 100 µM Cd^2+^. (**B**) Box plot shows aggregated data of Ca^2+^ current before (control) and after s-cal14.2b and Cd^2+^ blockade. (**C**) Average increase in Ca^2+^ current amplitude with s-cal14.2b and Cd^2+^ blockade. (**D**) Summary of time-constant (τ) of the increase and recovery of Ca^2+^ current amplitude with s-cal14.2b-treatment. Thus, s-cal14.2b significantly augmented calcium influx through Ca_V_ 1.2/1.3 channels. Data are presented as mean ± SE (*n* = 5). Statistical significance compared to control is indicated by * where *p* < 0.05.

Voltage-gated calcium channel currents (Cav1.2/1.3) were recorded using the patch-clamp technique in whole-cell configuration. The selection of rat pancreatic β-cells for electrophysiological recordings was based on a typical capacitance (i.e., 6–9 pF) and granular morphology. The s-cal14.2b peptide was applied to the pancreatic β-cells by microperfusion with 100 µM Cd^2+^ used to define the voltage-sensitive Ca^2+^ current (Figure 4A). Current amplitude was blocked by Cd^2+^ (in the grey area). Treatment with s-cal14.2b significantly increased the current amplitude through Cav1.2/1.3 channels by 35 ± 4%, as shown in a representative trace (Figure 3E) and time course (Figure 4A). Conversely, Cd^2+^ significantly blocked current amplitude by 98 ± 0.8% (Figure 4C). Time constant (τ) of the increase and (τ) recovery observed with s-cal14.2b was 21.17 ± 6 and 10.3 ± 4 s, respectively (Figure 4D). Intracellular free calcium concentration ([Ca^2+^]i) is a keystone in insulin secretion as voltage-gated calcium channels are the canonical entry for calcium influx. Thus, s-cal14.2b readily enhances Ca_V_1.3 conductance as a critical determinant for initiating and sustaining insulin secretion. Overall, along with complex mechanisms, an increase in [Ca^2+^]i is the primary insulin secretory signal in pancreatic β-cells in response to glycemia.

### 3.5. Glucose Tolerance Curve in Mice Model after s-cal14.2b Administration

**Figure 5:**
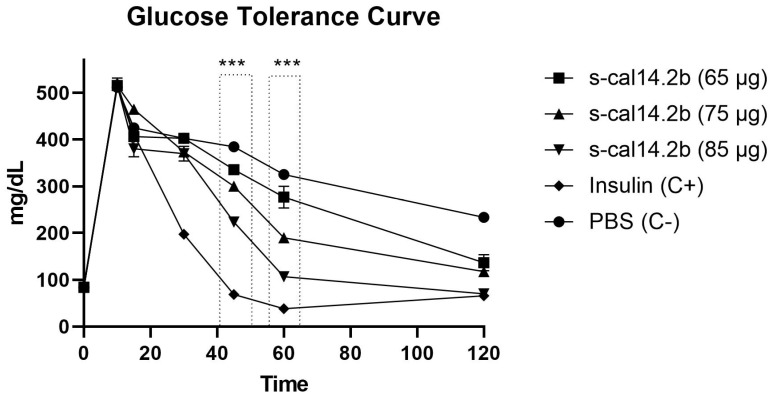
Glucose tolerance in mice following administration of s-cal14.2b. Three different treatments of s-cal14.2b (65, 75, and 85 μg/20 g body weight) were administered to mice resulting in a concentration-dependent effect of the syn-thetic peptide on glucose levels, when compared to the PBS control group. Modulation of glucose levels by the positive insulin control group (C+) began at 30 min when levels became significantly lower than the negative PBS control group (C−). By 45 min-post administration, blood glucose levels in the s-cal14.2b groups significantly decreased, compared to the PBS control group. By 120 min, the s-cal14.2b treatment group responded similarly to the positive insulin control group. Results are expressed mean ± SEM assays conducted in triplicate (*n* = 3). Statistical significance denoted by *** *p* < 0.001 between control positive and s-cal14.2b concentrations.

The blood glucose levels measured in mice revealed a dosage-dependent response to s-cal14.2b treatment. In Figure 5, administration of s-cal14.2b or insulin did not significantly change blood glucose levels compared to the PBS control group after five minutes. However, the insulin group displayed significantly lower blood glucose levels compared to the PBS and s-cal14.2b groups 30 min after administration. After 45 min, blood glucose levels significantly decreased in all of the s-cal14.2b treatment groups compared to the PBS control group. Furthermore, after 60 min, blood glucose levels in all s-cal14.2b groups were significantly different to each other, revealing a dosage-dependent response to s-cal14.2b. At 120 min, the mice showed no signs of respiratory distress associated with glycemia.

### 3.6. Biodistribution of Radiolabeled s-cal14.2b in Wistar Rats

**Figure 6:**
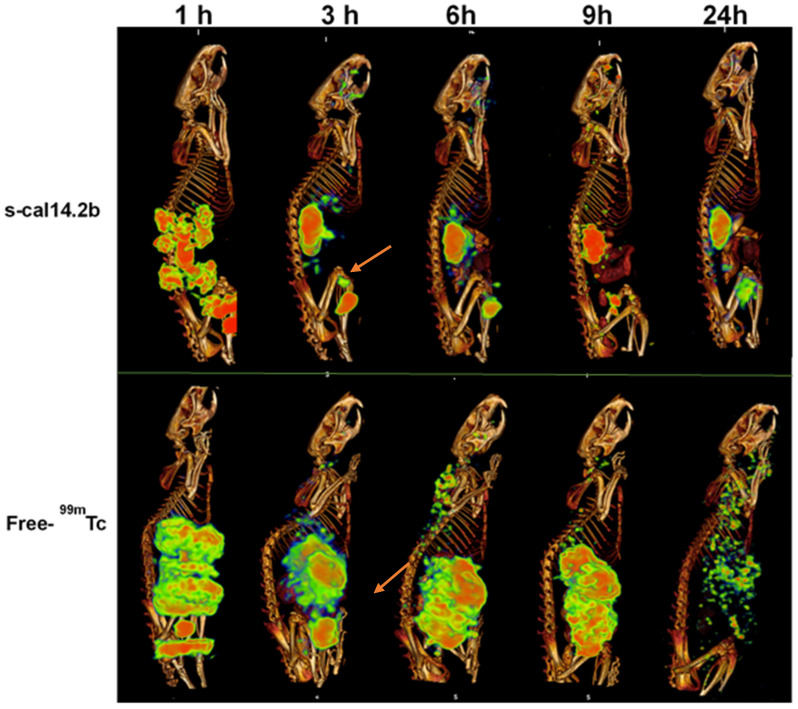
Biodistribution of radiolabeled s-cal14.2b in rats. The s-cal14.2b synthetic peptide was radiolabeled with 99mTc and administered to rats by intraperitoneal injection. The majority of 99mTc-s-cal14.2b was observed in peripancreatic and pancreatic regions (shown by arrows) with clearance most likely by renal excretion (even though s-cal14.2b was still detected 24 h after administration). Free 99mTc shows a different pattern of biodistribution and clearance by 24 h post administration.

The s-cal14.2b peptide was radiolabeled directly with 99mTc producing an efficiency of 77 ± 2% (mean ± SD). Comparatively, human serum stability was 93 ± 3.01% at 1 h, 89 ± 0.66% at 3 h, 89 ± 0.34% at 6 h, 84 ± 0.14% at 12 h, and 81 ± 2.52% at 24 h (Figure 6). Based on this data, 99mTc-s-cal14.2b was deemed sufficiently stable for rat biodistribution studies.

Radiolabeled biodistribution analysis was performed in three different anatomic planes: transverse, coronal, and sagittal (Figure 6). After the first hour, 99mTc-s-cal14.2b was present at the site of injection as well in the kidneys, bladder, liver, pancreas, and be-tween lumbar vertebrae. After three hours, a uniform distribution of 99mTc-s-cal14.2b was observed in the kidneys, bladder, and salivary gland, with less 99mTc-s-cal14.2b present in liver. After six hours, 99mTc-s-cal14.2b accumulation was observed mainly in the kidneys, bladder, and liver. After 9 and 24 h post injection, less 99mTc-s-cal14.2b were observed with some accumulation evident in the kidneys and bladder (Figure 6, top panel). The control group, administered with unconjugated 99mTc, exhibited a normal distribution of free-99mTc in the stomach, salivary glands, thyroid, kidneys, and spleen (Figure 6, bottom panel). Overall, findings suggest that clearance of 99mTc-s-cal14.2b, as well as free 99mTc, is through renal excretion.

## 4. Discussion and Conclusions

In this study, the synthetic toxin cal14.2b (s-cal14.2b), derived from the venom of *C. californicus*, modulated insulin secretion in vitro and decreased blood glucose levels in vivo. In a glucose tolerance test in BALB/C mice, administration of s-cal14.2b decreased blood glucose after 120 min in a similar manner to recombinant insulin. Immunocytochemistry and electrophysiology revealed that s-cal14.2b possibly stimulates insulin secretion by pancreatic β-cells. The dosage-dependent, insulin-like effect of s-cal14.2b in this study suggests that this synthetic peptide could be a new drug candidate for treating T2DM, particularly in the early stages of this disease when pancreatic damage is limited [47,48]. The synthetic cal14.2b peptide is the first conopeptide (less than 20 amino acids) discovered that can modulate the secretion of insulin. Additionally, if pancreatic β-cell plasticity in response to s-cal14.2b is considered, insulin production may also be stimulated [49]. Taking the example of exendin 4 as a precedent to the usage of natural peptides as treatment of metabolic diseases and the promising results in human therapies [50], this conotoxin needs scale in type 2 diabetes in vitro model’s complexity as in 3D cell culture with pancreatic human cells to assess the tissue like penetration and evaluate the potential effect on autocrine control on human pancreatic cells [51].

An increase in intracellular Ca^2+^ concentration ([Ca^2+^]_i_) is a prerequisite for insulin secretion. Ca_V_1.2/1.3 L-type Ca^2+^ channels are the main entry to trigger insulin release in all pancreatic β-cells and insulin-secreting cell lines studied [52]. Even though this is the case, GLP-I (7–36) amide augments Ba^2+^ current through L-Type Ca^2+^ channels of rats’ pancreatic β-cells [53]. Classically, dihydropyridine (DHP)-sensitive Ca_V_1.2/1.3 channels are responsible for endocrine secretion [54]. Thus, excitation-secretion coupling of insulin release comprises triggering and modulation mechanisms [55]. Pro-releasing insulin agents should be able to act without altering physiological responses. Accordingly, electrophysiological, and biophysical methods have been proven to preserve homeostatic cellular functions to study ion channel currents. Thus, isolated pancreatic β-cells were efficiently recorded in primary culture conditions unveiling a significant enhancement of calcium current under s-cal14.2b. Our results reproduce and confirm the primary target on Ca_V_1.2/1.3 L-type DHP-sensitive Ca^2+^ channels [48]. There was also increased Ca^2+^ conductance and Ca^2+^ influx through these channels. They open with strong depolarizations and conduct Ba^2+^ better than Ca^2+^, so that we used Ba^2+^ as a charge carrier. Also, this finding in cultured single cells, which echoes those evaluated with in vitro toxicological parameters of s-cal14.2b as this compound lacks toxic effects on NIT-1 cell line. Furthermore, synthetic conotoxin s-cal14.2b demonstrates the versatility of native conotoxins bridging the gap and crossing the species barrier, thereby acting either on physiological or pathophysiological targets. Despite that related current research has centered its attention on neurological or cancer models, s-cal14.2b has proven its effectiveness on endocrinological models to stimulate insulin secretion, thus enabling glucose levels modulation, as supported by in vivo glucose tolerance curve without affecting the biodistribution as confirmed by radiolabeled s-cal14.2b. Cellular depolarization triggers the exocytosis of insulin granules as a result of Ca^2+^ entry with [Ca^2+^]i, mostly through L-type voltage dependent calcium channels [56,57]. This localized calcium influx results in both slow and fast insulin exocytosis through different mechanisms according to the preparatory configurations of SNARE complex [58]. Increasingly, a variety of channelopathies has been described nowadays, even though excitation-secretion coupling of insulin by Ca^2+^ influx is largely supported. Therefore, either genetic or acquired dysregulation of ion channels maybe present as the primary condition to develop metabolic derangements such as DM2. Restoring insulin release and plasmatic glucose levels should be the outstanding goal of any successful treatment. Undoubtedly, native, or synthetic conotoxins may be an option to find and develop novel therapeutic compounds. Insulin release is subject to several modulation mechanisms that exert the potentiation and inhibition responses of endogenous and exogenous stimuli in several ways [59,60,61]. Indeed, calcium entry in pancreatic β-cell calcium entry triggered through the closing of K_ATP_ channels (sulfonylureas) or modulated by pharmacological stimulation of GPCRs (GLP-1, exendin 4, DPP-4 inhibitors) remains a cornerstone of current therapeutic options for T2DM [62].

To explain the dose-dependent increase in glucose tolerance observed in Figure 5, we must consider the possible effects of s-cal14.2b. Cell cultures of NIT cells and primarily isolated pancreatic beta cells were performed. NIT cells used to assess s-cal14.2b in Figure 2 shows that in comparable glucose concentrations, only active s-cal14.2b elicited insulin release, while s-cal14.1a did not result in an insulin release. Their ability to induce insulin release in glucose concentrations was unable to elicit insulin release by themselves, revealing that the triggering pathway mediates this effect [55]. By performing whole-cell patch-clamp experiments, we were able to further dissect the possible mechanisms of action of s-cal14.2b. Internal solutions containing TEA blocked K_ATP_ channels [63] while TTX administration in the external solution blocked Na^+^ channels [64]. Thus, Ca^2+^ channel currents were isolated. In this conditions administration of s-cal14.2b resulted in an increase of the calcium current (Figure 3E). The onset of this effect takes some seconds to reach its peak and is partially washed out (Figure 4). This slow onset and partial washing of the effect indicates the activation of intracellular signals, therefore suggesting a metabotropic effect characteristically slower than an ionotropic effect [65,66]. Accordingly, s-cal14.2b action in [Ca^2+^]i must be due to a voltage-insensitive mechanism [48].

The synthetic peptide derived from *Californiconus californicus*, s-cal14.2b, modulates Ca^2+^ at betta pancreatic cells. An increase in intracellular Ca^2+^ concentration ([Ca^2+^]i) is a prerequisite for insulin secretion. s-cal14.2b readily enhances Ca_V_1.2/1.3 conductance as a critical determinant for initiating and sustaining insulin secretion, demonstrating an insulinotropic effect on in vitro and in vivo models and suggesting the possibility of moving forward in the assessment of this compound as a promising novel therapeutic compound to regulate insulin secretion in T2DM 3D models and in vivo as a preclinical trial.

## Data Availability

All data generated or analyzed during this study are included in the published article.

## References

[1] Danaei G., Finucane M.M., Lu Y., Singh G.M., Cowan M.J., Paciorek C.J., Lin J.K., Farzadfar F., Khang Y.H., Stevens G.A. (2011). National, regional, and global trends in fasting plasma glucose and diabetes prevalence since 1980: Systematic analysis of health examination surveys and epide-miological studies with 370 country-years and 2.7million participants. Lancet.

[2] International Diabetes Federation (2013). Diabetes Atlas.

[3] WHO 10 Facts about Diabetes. http://www.whoint/features/factfiles/diabetes/facts/en/index.html.

[4] American Diabetes Association (2013). Economic costs of diabetes in the U.S. in 2012. Diabetes Care.

[5] Breton M.C., Guenette L., Amiche M.A., Kayibanda J.F., Gregoire J.P., Moisan J. (2013). Burden of diabetes on the ability to work: A systematic review. Diabetes Care.

[6] McEwen L.N., Casagrande S.S., Kuo S., Herman W.H. (2017). Why Are Diabetes Medications So Expensive and What Can Be Done to Control Their Cost?. Curr. Diabetes Rep..

[7] Bhutani J., Bhutani S. (2014). Worldwide burden of diabetes. Indian J. Endocrinol. Metab..

[8] Sarwar N., Gao P., Seshasai S.R., Gobin R., Kaptoge S., Di Angelantonio E., Ingelsson E., Lawlor D.A., Selvin E., Stampfer M. (2010). Diabetes mellitus, fasting blood glucose concentration, and risk of vascular disease: A collaborative meta-analysis of 102 prospective studies. Lancet.

[9] Barquera S., Hernández-Alcaraz C., Jáuregui A., Medina C., Mendoza-Herrera K., Pedroza-Tobias A., Tolentino Mayo L., Guillen Pineda L.E., López-Ridaura R., Aguilar Salinas C.A. (2021). Diabetes awareness, treatment, and control among Mexico City residents. Diabetology.

[10] Weihrauch-Blüher S., Wiegand S. (2017). Risk factors and implications of childhood obesity. Curr. Obes. Rep..

[11] Genuth S., Alberti K.G., Bennett P., Buse J., Defronzo R., Kahn R., Kitzmiller J., Knowler W.C., Lebovitz H., Lernmark A. (2003). Expert Committee on the Diagnosis and Classification of Diabetes Mellitus. Follow-up report on the diagnosis of diabetes mellitus. Diabetes Care.

[12] American Diabetes Association (2021). Introduction: Standards of Medical Care in Diabetes—2021. Diabetes Care.

[13] American Diabetes Association (2013). Diagnosis and classification of diabetes mellitus. Diabetes Care.

[14] Lingvay I. (2017). Sodium glucose cotransporter 2 and dipeptidyl peptidase-4 inhibition: Promise of a dynamic duo. Endocr. Pract..

[15] Kalra S., Kesavadev J., Chadha M., Kumar G.V. (2018). Sodium-glucose Cotransporter-2 Inhibitors in Combination with Other Glucose-lowering Agents for the Treatment of Type 2 Diabetes Mellitus. Indian J. Endocrinol. Metab..

[16] Lajara R. (2019). Combination therapy with SGLT-2 inhibitors and GLP-1 receptor agonists as complementary agents that address multi-organ defects in type 2 diabetes. Postgrad. Med..

[17] Chaudhury A., Duvoor C., Reddy Dendi V.S., Kraleti S., Chada A., Ravilla R., Marco A., Shekhawat N.S., Montales M.T., Kuriakose K. (2017). Clinical review of antidiabetic drugs: Implications for Type 2 Diabetes Mellitus management. Front. Endocrinol..

[18] Westermeier F., Holyoak T., Asenjo J.L., Gatica R., Nualart F., Burbulis I., Bertinat R. (2019). Gluconeogenic enzymes in β-Cells: Pharmacological targets for improving insulin secretion. Trends Endocrinol. Metab..

[19] Khunti K., Gomes M.B., Pocock S., Shestakova M.V., Pintat S., Fenici P., Hammar N., Medina J. (2018). Therapeutic inertia in the treatment of hyperglycaemia in patients with type 2 diabetes: A systematic review. Diabetes Obes. Metab..

[20] DeFronzo R.A., Ratner R.E., Han J., Kim D.D., Fineman B.S., Baron A.D. (2005). Effects of exenatide (exendin-4) on glycemic control and weight over 30 weeks in metformin-treated patients with type 2 diabetes. Diabetes Care.

[21] Li Z., Zhou Z., Huang G., Hu F., Xiang Y., He L. (2013). Exendin-4 protects mitochondria from reactive oxygen species induced apoptosis in pancreatic beta cells. PLoS ONE.

[22] Yap M.K.K., Misuan N. (2019). Exendin-4 from Heloderma suspectum venom: From discovery to its latest application as type II diabetes combatant. Basic Clin. Pharmacol. Toxicol..

[23] Sanggaard K.W., Dyrlund T.F., Thomsen L.R., Nielsen T.A., Brøndum L., Wang T., Thøgersen I.B., Enghild J.J. (2015). Characterization of the gila monster (*Heloderma suspectum suspectum*) venom proteome. Data Br..

[24] Nathan D.M., Buse J.B., Davidson M.B., Ferrannini E., Holman R.R., Sherwin R., Zinman B. (2009). American Diabetes Association, European Association for Study of Diabetes. Medical management of hyperglycemia in type 2 diabetes: A consensus algorithm for the initiation and adjustment of therapy: A consensus statement of the American Diabetes Association and the European Association for the Study of Diabetes. Diabetes Care.

[25] Davies M.J., D’Alessio D.A., Fradkin J., Kernan W.N., Mathieu C., Mingrone G., Rossing P., Tsapas A., Wexler D.J., Buse J.B. (2018). Management of hyperglycemia in type 2 diabetes, (2018). A consensus report by the American Diabetes Association (ADA) and the European Association for the Study of Diabetes (EASD). Diabetes Care.

[26] Garber A.J., Abrahamson M.J., Barzilay J.I., Blonde L., Bloomgarden Z.T., Bush M.A., Dagogo-Jack S., DeFronzo R.A., Einhorn D., Fonseca V.A. (2019). Consensus statement by the American association of clinical endocrinologists and American college of endocrinology on the comprehensive type 2 diabetes management algorithm—2019 executive summary. Endocr. Pract..

[27] Kosiborod M., Cavender M.A., Fu A.Z., Wilding J.P., Khunti K., Holl R.W., Norhammar A., Birkeland K.I., Jørgensen M.E., Thuresson M. (2017). CVD-REAL Investigators and Study Group. Lower risk of heart failure and death in patients initiated on sodium-glucose cotransporter-2 inhibitors versus other glucose-lowering drugs: The CVD-REAL study (comparative effectiveness of cardiovascular outcomes in new users of sodium-glucose cotransporter-2 inhibitors). Circulation.

[28] Guillausseau P.J., Meas T., Virally M., Laloi-Michelin M., Médeau V., Kevorkian J.P. (2008). Abnormalities in insulin secretion in type 2 diabetes mellitus. Diabetes Metab..

[29] McGivern J.G. (2007). Ziconotide: A review of its pharmacology and use in the treatment of pain. Neuropsychiatr. Dis. Treat..

[30] Olivera B.M., McIntosh J.M., Cruz L.J., Luque F.A., Gray W.R. (1984). Purification and sequence of a presynaptic peptide toxin from *Conus geographus* venom. Biochemistry.

[31] Olivera B.M., Rivier J., Clark C., Ramilo C.A., Corpuz G.P., Abogadie F.C., Mena E.E., Woodward S.R., Hillyard D.R., Cruz L.J. (1990). Diversity of Conus neuropeptides. Science.

[32] Olivera B.M. (1997). *Conus* venom peptides, receptor and ion channel targets, and drug design: 50 million years of neuropharmacology. Mol. Biol. Cell.

[33] Biggs J.S., Watkins M., Puillandre N., Ownby J.P., Lopez-Vera E., Christensen S., Juárez-Moreno K., Bernaldez J., Licea-Navarro A., Corneli P.S. (2010). Evolution of Conus Peptide Toxins: Analysis of *Conus californicus* Reeve, 1844. Mol. Phylogenet. Evol..

[34] Puillandre N., Duda T.F., Meyer C., Olivera B.M., Bouchet P. (2015). One, four or 100 genera? A new classification of the cone snails. J. Molluscan Stud..

[35] Craig A.G., Jimenez E.C., Dykert J., Nielsen D.B., Gulyas J., Abogadie F.C., Porter J., Rivier J.E., Cruz L.J., Olivera B.M. (1997). A novel post translational modification involving bromination of tryptophan—identification of the residue, l-6-bromotryptophan, in peptides from *Conus imperialis* and *Conus radiatus* venom. J. Biol. Chem..

[36] Terlau H., Olivera B. (2004). *Conus* Venoms: A rich source of novel ion channel-targeted peptides. Physiol. Rev..

[37] Olivera B.M., Teichert R.W. (2007). Diversity of the neurotoxic *Conus* peptides: A model for concerted pharmacological discovery. Mol. Interv..

[38] Severgnini M., Sherman J., Sehgal A., Jayaprakash N.K., Aubin J., Wang G., Zhang L., Peng C.G., Yucius K., Butler J. (2012). A rapid two-step method for isolation of functional primary mouse hepatocytes: Cell characterization and asialoglycoprotein receptor-based assay development. Cytotechnology.

[39] Szot G.L., Koudria P., Bluestone J.A. (2007). Transplantation of pancreatic islets into the kidney capsule of diabetic mice. J. Vis. Exp..

[40] Hamaguchi K., Gaskins H.R., Leiter E.H. (1991). NIT-1, a pancreatic beta-cell line established from a transgenic NOD/Lt mouse. Diabetes.

[41] Wu Y.J., Wu Y.B., Fang Z.H., Chen M.Q., Wang Y.F., Wu C.Y., Lv M.A. (2019). Danzhi Jiangtang capsule mediates NIT-1 Insulinoma cell proliferation and apoptosis by GLP-1/Akt signaling pathway. Evid. Based Complement. Altern. Med..

[42] Najjar A., Alawi M., AbuHeshmeh N., Sallam A. (2014). A rapid, isocratic HPLC method for determination of insulin and its degradation product. Adv. Pharm..

[43] Zhang X., Yoon H.J., Kang M.G., Kim G.J., Shin S.Y., Baek S.H., Lee J.G., Bai J., Lee S.Y., Choi M.J. (2018). Identification and evaluation of cytotoxicity of peptide liposome incorporated citron extracts in an in vitro system. Int. J. Mol. Sci..

[44] Lee H.S., Jeong J., Lee K.J. (2009). Characterization of vesicles secreted from insulinoma NIT-1 cells. J. Proteome Res..

[45] Andrikopoulos S., Blair A.R., Deluca N., Fam B.C., Proietto J. (2008). Evaluating the glucose tolerance test in mice. Am. J. Physiol. Endocrinol. Metab..

[46] Geskovski N., Kuzmanovska S., Simonoska Crcarevska M., Calis S., Dimchevska S., Petrusevska M., Zdravkovski P., Goracinova K. (2013). Comparative biodistribution studies of technetium-99 m radiolabeled amphiphilic nanoparticles using three different reducing agents during the labeling procedure. J. Label. Comp. Radiopharm..

[47] Hiriart M., Vidaltamayo R., Sánchez-Soto M.C. (2001). Nerve and fibroblast growth factors as modulators of pancreatic beta cell plasticity and insulin secretion. Isr. Med. Assoc. J..

[48] de la Cruz L., Puente E.I., Reyes-Vaca A., Arenas I., Garduño J., Bravo-Martínez J., Garcia D.E. (2016). PIP2 in pancreatic β-cells regulates voltage-gated calcium channels by a voltage-independent pathway. Am. J. Physiol. Cell Physiol..

[49] Kahn S.E. (2001). The importance of β-cell failure in the development and progression of type 2 diabetes. Int. J. Clin. Endocrinol. Metab..

[50] Molina J., Rodriguez-Diaz R., Fachado A., Jacques-Silva M.C., Berggren P.O., Caicedo A. (2014). Control of insulin secretion by cholinergic signaling in the human pancreatic islet. Diabetes.

[51] Klonoff D.C., Buse J.B., Nielsen L.L., Guan X., Bowlus C.L., Holcombe J.H., Wintle M.E., Maggs D.G. (2008). Exenatide effects on diabetes, obesity, cardiovascular risk factors and hepatic biomarkers in patients with type 2 diabetes treated for at least 3 years. Curr. Med. Res. Opin..

[52] Henquin J.C. (2021). Paracrine and autocrine control of insulin secretion in human islets: Evidence and pending questions. Am. J. Physiol. Endocrinol. Metab..

[53] Hiriart M., Aguilar-Bryan L. (2008). Channel regulation of glucose sensing in the pancreatic β-cell. Am. J. Physiol. Endocrinol. Metab..

[54] Suga S., Kanno T., Nakano K., Takeo T., Dobashi Y., Wakui M. (1997). GLP-I (7–36) amide augments Ba^2+^ current through L-type Ca^2+^ channel of rat pancreatic beta-cell in a cAMP-dependent manner. Diabetes.

[55] Catterall W.A. (2011). Voltage-gated calcium channels. Cold Spring Harb. Perspect. Biol..

[56] Henquin J.C. (2000). Triggering and amplifying pathways of regulation of insulin secretion by glucose. Diabetes.

[57] Ashcroft F.M., Proks P., Smith P.A., Ammälä C., Bokvist K., Rorsman P. (1994). Stimulus-secretion coupling in pancreatic beta cells. J. Cell. Biochem..

[58] Gilbert M., Jung S.-R., Reed B.J., Sweet I.R. (2008). Islet oxygen consumption and insulin secretion tightly coupled to calcium derived from L-type calcium channels but not from the endoplasmic reticulum. J. Biol. Chem..

[59] Takahashi N., Hatakeyama H., Okado H., Noguchi J., Ohno M., Kasai H. (2010). SNARE Conformational changes that prepare vesicles for exocytosis. Cell Metab..

[60] Ashcroft F.M., Rorsman P. (1989). Electrophysiology of the pancreatic beta-cell. Prog. Biophys. Mol. Biol..

[61] Henquin J.C. (2004). Pathways in beta-cell stimulus-secretion coupling as targets for therapeutic insulin secretagogues. Diabetes.

[62] Rutter G.A., Pullen T.J., Hodson D.J., Martinez-Sanchez A. (2015). Pancreatic β-cell identity, glucose sensing and the control of insulin secretion. Biochem. J..

[63] American Diabetes Association (2021). 9. Pharmacologic Approaches to Glycemic Treatment: Standards of Medical Care in Diabetes—2021. Diabetes Care.

[64] Fatherazi S., Cook D.L. (1991). Specificity of tetraethylammonium and quinine for three K channels in insulin-secreting cells. J. Membr. Biol..

[65] Donatsch P., Lowe D.A., Richardson B.P., Taylor P. (1977). The functional significance of sodium channels in pancreatic beta-cell membranes. J. Physiol..

[66] Silbering A.F., Benton R. (2010). Ionotropic and metabotropic mechanisms in chemoreception: ‘chance or design’?. EMBO Rep..

